# Anti-Tumor Effects of Biomimetic Sulfated Glycosaminoglycans on Lung Adenocarcinoma Cells in 2D and 3D In Vitro Models

**DOI:** 10.3390/molecules25112595

**Published:** 2020-06-03

**Authors:** Nada Al Matari, George Deeb, Hiba Mshiek, Ansam Sinjab, Humam Kadara, Wassim Abou-Kheir, Rami Mhanna

**Affiliations:** 1 Department of Biomedical Engineering, Maroun Semaan Faculty of Engineering and Architecture, American University of Beirut, Beirut 1107 2020, Lebanon; nma77@mail.aub.edu (N.A.M.); gdd01@mail.aub.edu (G.D.); 2 Department of Anatomy, Cell Biology, and Physiological Sciences, Faculty of Medicine, American University of Beirut, Beirut 1107 2020, Lebanon; hm99@aub.edu.lb; 3 Department of Translational Molecular Pathology, The University of Texas MD Anderson Cancer Center, Houston, TX 77030, USA; asinjab@mdanderson.org (A.S.); hkadara@mdanderson.org (H.K.)

**Keywords:** glycosaminoglycans, lung adenocarcinoma, sulfated alginates, biomimetic, cancer stem cells

## Abstract

Lung cancer development relies on cell proliferation and migration, which in turn requires interaction with extracellular matrix (ECM) components such as glycosaminoglycans (GAGs). The mechanisms through which GAGs regulate cancer cell functions are not fully understood but they are, in part, mediated by controlled interactions with cytokines and growth factors (GFs). In order to mechanistically understand the effect of the degree of sulfation (DS) of GAGs on lung adenocarcinoma (LUAD) cells, we synthesized sulfated alginate (AlgSulf) as sulfated GAG mimics with DS = 0.0, 0.8, 2.0, and 2.7. Human (H1792) and mouse (MDA-F471) LUAD cell lines were treated with AlgSulf of various DSs at two concentrations 10 and 100 µg/mL and their anti-tumor properties were assessed using 3-(4,5-dimethylthiazol-2-yl)-2,5-diphenyltetrazolium bromide (MTT), trypan blue exclusion, and wound healing assays for 2D models and sphere formation assay for the 3D model. The proliferation and number of live MDA-F471 cells at the concentration of 100 µg/mL decreased significantly with the increase in the DS of biomimetic GAGs. In addition, the increase in the DS of biomimetic GAGs decreased cell migration (*p* < 0.001 for DS = 2.0 and 2.7 compared to control) and decreased the diameter and number of spheres formed (*p* < 0.001). The increased DS of biomimetic GAGs attenuated the expression of cancer stem cell (CSC)/progenitor markers in the 3D cultures. In conclusion, GAG-mimetic AlgSulf with increased DS exhibit enhanced anti-proliferative and migratory properties while also reducing growth of *KRAS*-mutant LUAD spheres in vitro. We suggest that these anti-tumor effects by GAG-mimetic AlgSulf are possibly due to differential binding to GFs and consequential decreased cell stemness. AlgSulf may be suitable for applications in cancer therapy after further in vivo validation.

## 1. Introduction

Cancer is the leading cause of death worldwide with approximately 9.6 million cancer-related deaths in 2018 [1]. Lung cancer is the second most diagnosed cancer after keratinocyte carcinoma (17 to 19% of all newly diagnosed cancers), and the leading cause of cancer deaths (19% of cancer deaths) [2,3]. Histologically, lung cancer is classified into one of two major histological types: small cell lung cancer and non-small cell lung cancer (NSCLC), contributing to about 15% and 85% of all lung cancer incidences, respectively [4,5]. NSCLC can be further histologically categorized into squamous-cell carcinomas, lung adenocarcinomas (LUAD), and large-cell lung carcinomas. LUADs are thought to be mutually exclusively driven by epithelial growth factor receptor (*EGFR*)-a dependent signaling and Kirsten rat sarcoma viral oncogene homolog (*KRAS*) pathway [6]. Somatic activating mutations in *KRAS* are the most prevalent oncogenic driver mutations in LUAD accounting for 25–30% of all mutations and are tightly associated with smoking [6,7]. Compared to other solid tumors, *KRAS*-mutant LUAD, even when diagnosed at early stages in its development, exhibits dismal prognosis and poor response to systemic or targeted therapy. While immune-based therapy has shown promising results, many *KRAS*-mutant LUADs still show poor to modest responses. The dismal prognosis of *KRAS*-mutant LUAD is thought to be, in part, due to genomic heterogeneity of *KRAS*-mutant LUADs (i.e., disparate co-occurring driver mutations). Thus, alternative therapeutic approaches are urgently warranted for early clinical management of this aggressive malignancy [8].

The extracellular matrix (ECM) is a dynamic tissue-specific structure made of several macromolecular components and fibers such as proteoglycans, proteins, and glycosaminoglycans (GAGs). The ECM provides mechanical support for cells and induces signals that regulate their behavior. The ECM plays a role in proliferation, growth, homeostasis, differentiation, and morphogenesis of cells [9,10]. At the cellular level, the ECM allows the binding of growth factors (GFs) and interacts with cells via cell-surface receptors [11]. Several methods to emulate the ECM to study its effects on the growth of normal, pathogenic, and cancerous cells have been proposed using hydrogels, coatings, and plates treated with fibroblasts [12]. One method to achieve ECM mimicry is using Matrigel™ coatings. Matrigel™ is suitable for tumor cell growth since it enhances angiogenesis, prevents tumor cell apoptosis, and supports 3D cell growth [13,14].

GAGs are composed of repeating disaccharide units with various degrees of acetylation, sulfation, and different bonding patterns. There are six types of GAG families; heparan sulfate, heparin, chondroitin sulfate, keratan sulfate, hyaluronic acid, and dermatan sulfate [15]. GAGs differ by their molecular mass, density, and biological functions, making them tissue-specific. All GAGs, except hyaluronic acid, are sulfated and bind to transmembrane protein cores by a serine residue. GAGs act as receptors and/or activators in the ECM and have various roles in homeostasis, angiogenesis, cell adhesion, protein secretion, gene transcription, and cell differentiation [16]. In pathological conditions, such as inflammation and cancer, GAGs are altered [15,16,17,18,19]. The role of GAGs in lung cancer is not properly understood due to their heterogeneous distribution within the ECM, the difficulty and high cost of purifying homogenous native GAGs, and the lack of suitable biomimetic molecules to perform systematic investigations.

GAGs selectively bind to several GFs, such as fibroblast growth factor-1 (FGF1) and FGF2. Due to its affinity with proteins, heparin interacts with the ECM and modulates cell signaling in wound healing, angiogenesis, and inflammatory responses [16,18,20,21,22,23]. Heparin can bind and concentrate GFs at its N-terminal, thereby promoting angiogenesis or hindering it by preventing the diffusion of GFs to cells [19]. Preventing angiogenesis in the tumor is one strategy to inhibit tumor growth. Heparin has also been shown to reduce the metastasis of lung, pancreatic and breast cancer tumors [24,25]. In addition, low molecular weight heparin affected cancer stem cell (CSC)-like LUAD by reducing their chemo-resistance [26]. In order to mimic the effects of sulfated GAGs, we manufactured heparin-mimetic sulfated alginate (AlgSulf) with controlled degrees of sulfation (DS) [27,28,29,30].

Alginate is a naturally occurring polysaccharide that is extracted from brown algae or Gram-negative bacteria’s exocellular polysaccharides and is structurally similar to heparin and GAGs in general [31,32]. Alginates are naturally non-sulfated but their carboxyl groups can be sulfated to mimic sulfated GAGs [30,31]. Various techniques allow controlling the DS of alginates using chlorosulfonic acid in formamide, pyridine, and carbodiimide-H_2_SO_4_ [30,33,34,35,36]. The DS of the synthesized alginate is the number of sulfate groups per one disaccharide unit; it ranges from 0 (no sulfation) to 4 (full sulfation of the free hydroxyl groups of the alginate). AlgSulf has been shown to promote the proliferation of chondrocytes and maintain the cartilage phenotype [37]. AlgSulf also possesses heparin-mimetic anticoagulant and anti-inflammatory properties [27,30,35,37,38].

We hypothesized that the DS of sulfated GAGs will affect the growth of LUAD cells in 2D and 3D in vitro cultures. To test this hypothesis, we used AlgSulf as a GAG mimic and assessed the effect of AlgSulf*_n_* (in which *n* represents the DS) treatment on *KRAS*-mutant LUAD cell proliferation using human and mouse models.

## 2. Results

### 2.1. Effect of the Biomimetic Sulfated GAGs on H1792 and MDA-F471 Metabolic Activity in 2D Cultures

**Figure 1:**
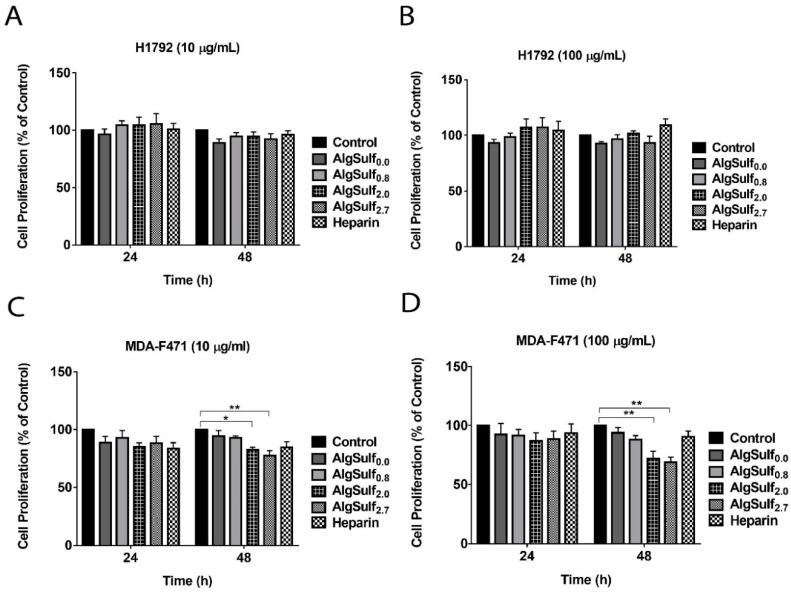
The effect of the DS of biomimetic sulfated GAGs on the activity of human (H1792) and murine (MDA-F471) LUAD cell lines. The metabolic activity of (**A**) H1792 treated with 10 µg/mL of AlgSulf*_n_* (**B**) H1792 treated with 100 µg/mL of AlgSulf*_n_* (**C**) MDA-F471 treated with 10 µg/mL of AlgSulf*_n_* and (**D**) MDA-F471 treated with 100 µg/mL of AlgSulf*_n_*. For each graph, heparin was added at the same concentration of the polysaccharides as positive controls, and untreated cells (media only) were used as a negative control. Results are expressed as the percentage of the treated group compared to control after 24 h and 48 h of culture. Data represent the average of five independent experiments and are reported as mean ± SEM (* *p* < 0.05; ** *p* < 0.01) using two-way ANOVA followed by Bonferroni’s multiple comparison test.

The effects of the increase in the DS of biomimetic GAGs on H1792 and MDA-F471 cell proliferation and activity were assessed using 3-(4,5-dimethylthiazol-2-yl)-2,5-diphenyltetrazolium bromide (MTT) assay. Two-way ANOVA followed by Bonferroni’s multiple comparison test showed that the metabolic activity of H1792 cells was not significantly affected by the DS of alginates supplemented at 10 or 100 µg/mL (Figure 1A,B). On the other hand, the activity of MDA-F471 cells treated with 10 µg/mL AlgSulf of DS = 2.0 and DS = 2.7 (AlgSulf_2.0_ and AlgSulf_2.7_) showed a significant decrease to 82% and 77% compared to untreated cells after 48 h of incubation (*p* = 0.025 and *p* = 0.002) respectively (Figure 1C). MDA-F471 cells treated with 100 µg/mL of AlgSulf_2.0_ and AlgSulf_2.7_ showed a significant decrease in the metabolic activity to 72% and 68% of untreated controls after 48 h (*p* = 0.005 and *p* = 0.002), respectively (Figure 1D).

### 2.2. The Increase in the DS of Biomimetic GAGs Reduces the Viability of H1792 and MDA-F471 LUAD Cell Lines in 2D Cultures

**Figure 2:**
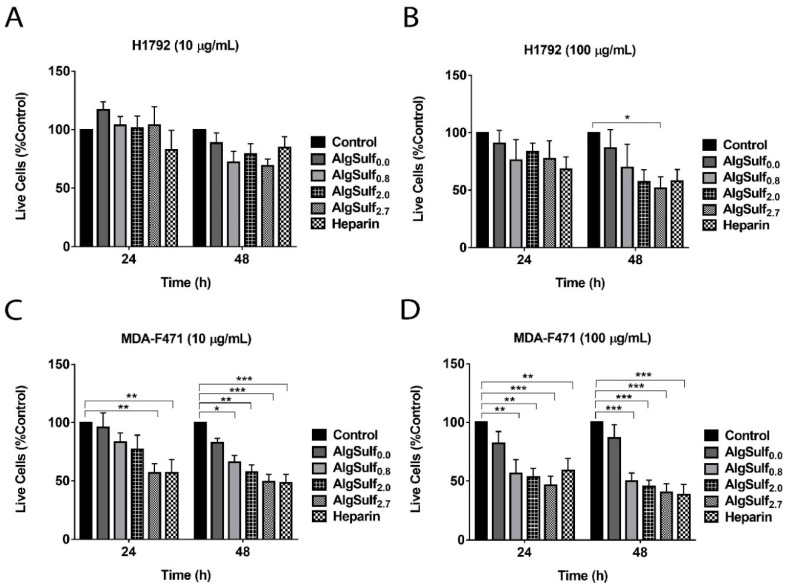
The effect of the DS of biomimetic sulfated GAGs on the viability of human and murine LUAD cell lines (**A**) H1792 treated with 10 µg/mL of AlgSulf*_n_*, (**B**) H1792 treated with 100 µg/mL of AlgSulf*_n_*, (**C**) MDA-F471 treated with 10 µg/mL of AlgSulf*_n_*, and (**D**) MDA-F471 treated with 100 µg/mL of AlgSulf*_n_* assessed using a trypan blue exclusion assay. For each graph, heparin was added at the same concentration of the polysaccharides as a positive control, and untreated cells (media only) were used as a negative control. Results are expressed as the percentage of the treated group compared to the control after 24 h and 48 h of culture. Data represent the average of five independent experiments and are reported as mean ± SEM (* *p* < 0.05; ** *p* < 0.01; *** *p* < 0.001) using two-way ANOVA followed by Bonferroni’s multiple comparison test.

The effect of the increase in the DS of the biomimetic sulfated GAGs on the viability of H1792 and MDA-F471 cells was assessed using trypan blue exclusion assay. The viability of H1792 cells at 10 µg/mL was not significantly affected by AlgSulf*_n_* treatment, as shown by two-way ANOVA (Figure 2A). H1792 cells treated with 100 µg/mL AlgSulf_2.7_ showed a significant decrease to 52% of the untreated cells after 48 h of incubation (*p* = 0.039) as shown by two-way ANOVA followed by Bonferroni’s multiple comparison test (Figure 2B). Although only AlgSulf_2.7_ had a significant effect on H1792 live cell count, a trend of decrease in cell numbers was clearly visible at 24 h and 48 h for all DSs. Treatment of MDA-F471 cells only with AlgSulf_2.7_ and heparin at 10 µg/mL caused a significant decrease in the percentage of viable cells after 24 h (*p* < 0.01), while after 48 h, all the sulfated materials showed a significant decrease (Figure 2C). The decrease in cell numbers upon treatment with AlgSulf*_n_* at a concentration of 100 µg/mL was also clearly observed on the MDA-F471 cells at all the DSs after 24 h (*p* < 0.01) and 48 h (*p* < 0.001) (Figure 2D).

### 2.3. The Increase in the Sulfation of Biomimetic GAGs Inhibits the Migratory Abilities of LUAD Cells

**Figure 3:**
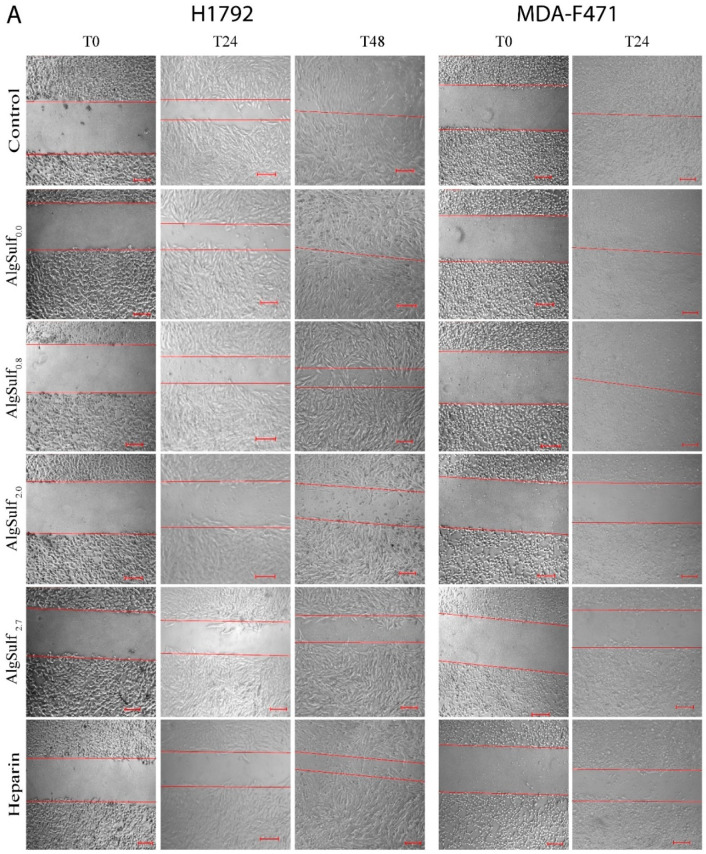

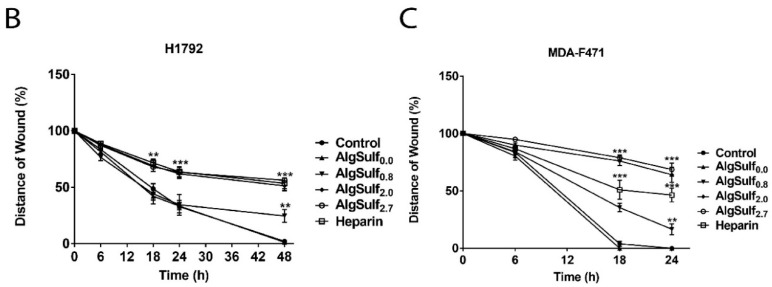
The effect of the DS of biomimetic sulfated GAGs on the migratory abilities of human (H1792) and murine (MDA-F471) LUAD cell lines. A scratch was made on a 12-well plate of confluent H1792 and MDA-F471 cells using a 200 µL micropipette tip and images were taken at T = 0, 6, 18, 24, and 48 h. (**A**) Representative bright-field images of H1792 and MDA-F471 wounds with different AlgSulf*_n_*. Results are expressed as the percentage of the wound distance in each group compared to its condition at T = 0 h for (**B**) H1792 and (**C**) MDA-F471 treated with AlgSulf*_n_*. Untreated cell layers were used as negative controls and heparin was used as a sulfated GAG positive control. Data represent the average of three independent experiments and are reported as mean ± SEM (** *p* < 0.01; *** *p* < 0.001) using two-way ANOVA followed by Bonferroni’s multiple comparison test. Scale = 100 µm.

The effect of the increase in the DS of biomimetic sulfated GAGs on the migratory properties of both cell lines was studied using a wound healing/scratch assay (Figure 3). Two-way ANOVA followed by Bonferroni’s multiple comparison test revealed that the increase in the DS of alginates, AlgSulf_2.0_ and AlgSulf_2.7_, significantly suppressed the migration of H1792 and MDA-F471 cells (*p* < 0.001), by 50% and 65%, respectively, as compared to the untreated cells in which the wound closed after 48 h and 24 h, respectively (Figure 3A–C). The data revealed that the increase in the DS of the biomimetic GAGs reduced the migratory abilities of LUAD cells. Figure S4 shows the progression of wound closure at all time points.

### 2.4. The Increase in the DS of AlgSulf_n_ Abolished the Sphere-Forming Abilities of Human and Murine LUAD Cells

**Figure 4:**
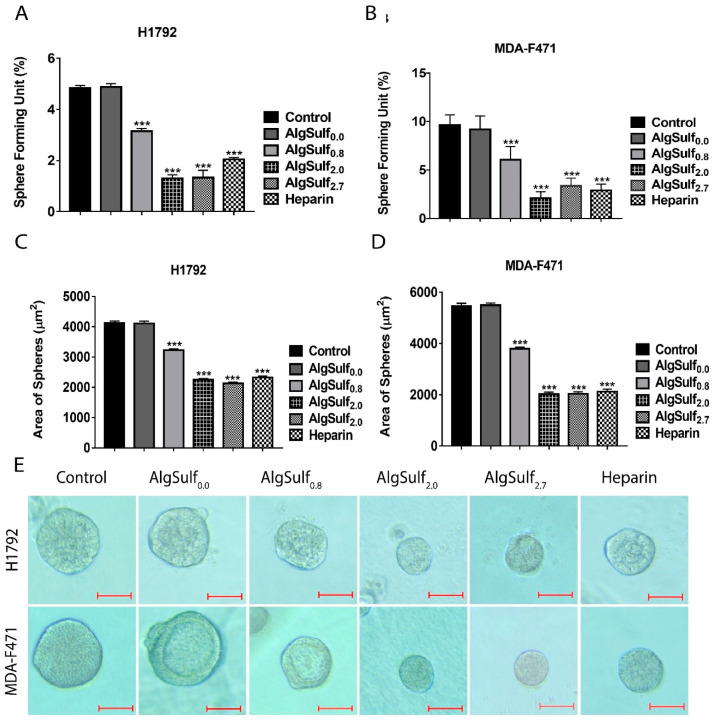
The effect of the DS of biomimetic sulfated GAGs on the sphere-forming capacities of human and murine LUAD cell lines. (**A** and **B**) Quantification of the sphere-forming unit (SFU) for H1792 and MDA-F471 spheres at G2 under different treatment conditions. (**C** and **D**) Quantification of the average area (µm^2^) of 30 spheres at G2. (**E**) Representative bright-field images of H1792 and MDA-F471 spheres at G2 with different DS of biomimetic GAGs. Data represent the average of three independent experiments and are reported as mean ± SEM (*** *p* < 0.001) using one-way ANOVA followed by Bonferroni’s multiple comparison test. Scale = 50 µm.

The sphere-forming capacity and cancer stem/progenitor’s growth were studied by culturing single-cell suspensions of H1792 and MDA-F471 on Matrigel™ for seven days (Figure 4). The spheres obtained from both cell lines at generation 2 (G2) were visualized under the Axiovert inverted microscope (Figure 4E). One-way ANOVA indicated that the increase in the DS of AlgSulf*_n_* resulted in a significant decrease in the sphere-forming unit (SFU) and the size of spheres formed of both cell lines with *p* < 0.001 (Figure 4A–D). Similar to heparin, AlgSulf_2.0_ and AlgSulf_2.7_ showed a significant decrease by almost 60% in the SFU in both cell lines. However, treatment with AlgSulf_0.0_ (non-sulfated controls) showed no decrease in the SFU and size of the spheres compared to non-treated controls. Remarkably, the increase in the DS of biomimetic GAGs exhibited more potent inhibitory effects on the proliferation of 3D-culture models than on 2D-culture models.

## 3. Discussion

Tumor development is mainly caused by genetic mutations leading in many cases to dysfunctionality in the ECM and its components such as GAGs. Several studies have shown the importance of sulfated GAGs in cellular activities, such as binding to GFs, angiogenesis, and anticoagulation [20,23,39]. Sulfated GAGs like heparin have been shown to induce tumor suppression in pancreatic cancer, metastatic breast cancer, and LUAD [24,25]. Moreover, expression of the heparin degrading endosulfatase HSulf-1, which diminishes the sulfation of cell-surface heparan sulfate proteoglycans, was found to be downregulated in breast, renal, pancreatic, hepatic, and ovarian cancer cells compared to normal cells [24,25,40]. Notably, re-expression of HSulf-1 resulted in reduced proliferation of ovarian cancer cells possibly by reducing GF binding [40]. The addition of exogenous heparin-mimetic molecules used in the current study was expected to shed GFs from LUAD cells, therefore, reducing their proliferation and migration as validated in the 2D and 3D culture systems of the current study. Not only were sulfated GAGs found to have modulatory effects on differentiated cancer cells but they were also found to target CSCs by promoting the degradation of ABCG2 protein, thus affecting signaling pathways and decreasing CSCs chemo-resistance, which is in agreement with the results of the current study [26].

Systematic studies on the impact of GAG sulfation on cancer cell growth are of high importance to decipher the mechanisms underlying cancer development and to develop effective therapies. These studies require the use of GAGs with homogenous structures, molecular weights, and sulfation patterns. However, the isolation and purification of homogenous sulfated GAGs is complicated and expensive. Sulfated polysaccharides have previously shown anti-proliferative and anti-migratory effects on cancerous cells cultured in 2D, which is in agreement with our findings [41,42]. Alginate is a linear polysaccharide and has the same backbone as GAGs with hydroxyl groups that can be sulfated using different sulfating agents, such as SO_3_/pyridine and HClSO_3_ acid formamide [27,28,29,31]. Synthesized sulfated alginates mimic sulfated GAGs by binding to GFs [36]. GF binding is critical for multiple cellular events, including proliferation, differentiation, and migration. This makes sulfation pivotal for normal cell growth, for example in wound healing and regeneration, but also for cancer cell growth and metastasis [29,36,43]. Biomimetic sulfated GAGs also showed proliferative effects on normal chondrocytes [36] and neural cell lines [43] possibly through increased GF binding. It is worth noting that our current and previous studies [36,43] assessed the role of the average DS of AlgSulf*_n_* treatment groups, which comprise a heterogeneous mixture of chains with a distribution of molecular weights, DSs, and positions of sulfate moieties (regioselectivity) that might have different individual contributions to cell behavior.

We assessed the anti-tumorigenic effects of biomimetic sulfated GAGs on two LUAD cell lines with a mutation on *KRAS* oncogene. The MTT assay used to assess the effects of biomimetic sulfated GAGs on the metabolic activity and proliferation of H1792 and MDA-F471 cell lines revealed no statistically relevant effects on the human *KRAS*-mutant driven LUAD, while it showed a significant effect on MDA-F471 cells after 48 h of treatment only with the high DS biomimetic GAGs AlgSulf_2.0_ and AlgSulf_2.7_. Moreover, despite a clear trend of decrease in H1792 live cells upon treatment with AlgSulf*_n_* as shown by the trypan blue exclusion assay, only those treated with 100 µg/mL of AlgSulf_2.7_ at 48 h showed a significant decrease. However, MDA-F471 viability decreased upon treatment with both concentrations of 10 µg/mL and 100 µg/mL exhibiting increased growth inhibition with increased DS of the AlgSulf*_n_*. This indicates that the increase in the sulfation of biomimetic GAGs has limited proliferative effects on 2D-cultured human LUAD cells, but has potent anti-proliferative effects on murine LUAD in a dose-dependent manner for the studied time-points. It is possible that human LUAD cells are more sensitive to dimensionality compared to murine; this hypothesis is supported by the significant response of human LUAD cells to treatment in 3D unlike 2D. The effects of AlgSulf*_n_* on the murine LUAD cell line were consistent with previous studies showing that sulfated polysaccharides have anti-proliferative effects on carcinoma cells [26,41,42]. Furthermore, we showed using a wound healing/ scratch assay that the migration of 2D cultured *KRAS*-mutant human and murine LUAD cells decreased when cells were exposed to biomimetic sulfated GAGs. The findings are consistent with previous studies, which showed that native sulfated GAGs reduce the migratory abilities of cancerous cells [41,42].

Finally, we determined the effects of the increase in the DS of biomimetic GAGs on the CSC population of H1792 and MDA-F471 cell lines, using a 3D sphere-forming assay. Cells cultured as 3D spheroid models were previously shown to better respond to administered drugs and to offer closer resemblance of the in vivo physiology [44,45]. Moreover, Riedl et al. showed that 3D sphere-forming assays are more representative of the in vivo response to anti-cancer drugs and may in cases be contradictory to results of 2D models [45]. Targeting CSCs is one of the aims of targeted cancer therapy given the evidence that supports the role of CSCs in tumorigenesis and tumor re-occurrence [46,47]. Based on a previous study from our team that showed the significant expression of CSC-properties in H1792 and MDA-F471 spheres at G2 and G5 [48], we analyzed the sphere-forming potentials of AlgSulf*_n_*-treated LUAD cells at G2. Our results verified that the increase in the DS of AlgSulf*_n_* significantly decreased the sphere formation capacity of human and murine LUAD cells where the SFU and the size of spheres at G2 decreased. QRT-PCR was performed as per the conditions detailed in Tables S1–S3. In addition, the qRT-PCR preliminary results of the cultivated spheres (*n* = 1) showed that the increase in the sulfation of biomimetic GAGs led to the decrease in the stemness properties of CSCs by down-regulation of some CSCs’ markers (Figure S5) [49,50,51,52,53,54,55,56]. Results showed downregulation of *ALDH1A1* and *ALDH3A1* in H1792 cells and *Aldh1a1* in MDA-F471 cells, which are known to be overexpressed in *KRAS*-mutant LUAD CSCs, the downregulation of *CCL20* in H1792 and MDA-F471, which plays a major role in the growth and metastasis of lung cancer cells, and the downregulation of *Alcam* and *Tnf* in MDA-F471, epithelial cell marker and proinflammatory cytokine, respectively [48]. These findings are consistent with other studies that stated the effects of anti-tumor agents on the CSC population using sphere-forming assay [46,47,48,49,50,57,58]. Furthermore, they motivate further investigations to determine the ability of AlgSulf*_n_* to target and affect CSCs growth and the pathway that is affected by the AlgSulf*_n_*.

Our study showed that the murine cell line is more sensitive to the sulfated biomimetic GAGs than the human cell line. This sensitivity may be because the murine cell line was developed from a lung adenocarcinoma in a mouse exposed to tobacco carcinogen, which made this cell line more aggressive. Another possible cause is the mutational landscape in the murine tumor cells, MDA-F471 cells carry *Kras* G12D variants, *Kras* gene amplification and very high somatic mutation burden while the H1792 cell line carries *KRAS* G12C variant and other co-occurring driver mutations as well as lower somatic mutation burden compared to the murine cell line [48]. These genomic differences may also extend to GF/receptor signaling pathways between the human and murine cells and thus, their anti-proliferative and migratory responsiveness to the sulfated biomimetic GAGs in monolayer and sphere assays [57].

Like cancer cells, normal cells require GF binding for proliferation; therefore, sulfation may be used to increase growth factor binding and enhance the growth of normal cells. We and others have shown earlier that increasing the sulfation of biomimetic sulfated GAGs enhances the growth of chondrocytes [36,59], neural cell lines [43], and fibroblasts [60], which is in contradiction with the finding of the current study that showed a decrease in cell growth. The discrepancy in results between the current study and the previous ones can be explained by the fact that previous studies used sulfated polysaccharides stabilized to a substrate whether in 2D or 3D unlike the current study where AlgSulf*_n_* were added in solution [36,43]. Sulfated polysaccharides used in previous studies were sterically less mobile while in the current study they might encapsulate growth factors, making them unavailable for GF receptors on the cell surface. We observed that the AlgSulf did not affect the morphology of the cells (Figure S2). To validate that the treatment modalities do not inhibit the growth of normal cells, we assessed the growth of normal prostate cells (RWPE1) treated with AlgSulf_2.7_ [59,61]. RWPE1 cells treated with AlgSulf_2.7_ showed no significant change in number of live cells as shown by the trypan blue exclusion assay (Figure S3). This might be because normal cells are less dependent on GFs than cancerous cells. Thus, the increase in the sulfation of biomimetic GAGs does not have an antiproliferative effect on normal epithelial cells.

## 4. Materials and Methods

### 4.1. Sulfated Alginate Preparation and Characterization

Synthesis of AlgSulf*_n_* was performed as previously described by Mhanna et al. [36]. First, 2 g of 5 mmol alginate (Sigma Aldrich, St. Louis, MO, USA) were dissolved in 400 mL of water and 40 g of DOWEX Marathon C ion exchanger (Sigma Aldrich, St. Louis, MO, USA), which had previously been charged with 40 g of tetrabutyl ammonium bromide (Sigma Aldrich, St. Louis, MO, USA). The mixture was left to stir overnight, then filtered and lyophilized. The resulting alginate tetrabutyl ammonium salt was then suspended in dry dimethylformamide (Sigma Aldrich, St. Louis, MO, USA). To produce alginates with varying DSs, different quantities of SO_3_/pyridine (Sigma Aldrich, St. Louis, MO, USA) per disaccharide repeating unit were added; a five-fold excess for DS 0.8, a nine-fold excess for DS 2.0, and a fifteen-fold excess for a DS of 2.7. The mixture was then stirred at room temperature for one hour. The resulting cloudy solution was precipitated with acetone, its pH raised to 12 with NaOH for 10 min, and then neutralized. The subsequent precipitate was filtered, dissolved in water, purified by dialysis, and lyophilized. The DS of the resulting products was determined by estimating the sulfur content using an automatic elemental analyzer (CHNS-932, Leco, Moenchengladbach, Germany). The schematic of the synthesis process is depicted in Figure S1.

### 4.2. Cell Culture and Treatment

The human *KRAS*-mutant LUAD (H1792) (ATCC, Manassas, VA, USA) and murine *Gprc5a^−/−^ Kras*-mutant LUAD (MDA-F471) [62] cell lines were maintained in DMEM F-12 medium (Sigma-Aldrich, St. Louis, MO, USA) supplemented with 10% fetal bovine serum (FBS) (Sigma-Aldrich, St. Louis, MO, USA), 1% penicillin-streptomycin antibiotics (Lonza, Basel, Switzerland), a humidified incubator at 37 °C with 5% CO_2_, and 5 μg/mL Plasmocin Prophylactic (InvivoGen, San Diego, CA, USA). In addition, we used RWPE1 (ATCC, Manassas, MO, USA), normal epithelial prostatic human cells, to confirm that there is no effect of the treatment on normal epithelial cells RWPE1 cells were cultured in keratinocyte serum-free medium (GIBCO, Thermo Fisher Scientific, Waltham, MA, USA) supplemented with 0.004% recombinant human epithelial growth factor (rhEGF) and incubated in a humidified incubator (5% CO_2_) at 37 °C. For passaging, cells were enzymatically dissociated using trypsin/EDTA (Sigma-Aldrich, St. Louis, MO, USA), until a maximum of 25 passages.

Throughout the study, different treatments were used to assess the effects of biomimetic sulfated GAGs on the anti-tumorigenic properties of LUAD cells. The AlgSulf*_n_* was produced following the procedure developed by Mhanna and his coworkers [36]. The treatments were dissolved in serum-free DMEM F-12 medium then filtered using cellulose acetate syringe filters, Abluo, 0.22 Micron, 25 mm, 50/Pk (GVS). The DSs of the alginates were 0.0, 0.8, 2.0, and 2.7, in addition to heparin (Sigma-Aldrich, St. Louis, MO, USA) extracted from the porcine intestinal mucosa, which acted as a sulfated GAG (positive control). The untreated wells in which we added only media acted as negative controls, while the vehicle wells were treated with a volume of serum-free media the same as that of added treatment.

### 4.3. Evaluation of Cell Morphology

Cells were plated in 24-well plates at the density of 20,000 and 30,000 cells/well for MDA-F471 and H1792, respectively, and incubated overnight at 37 °C and 5% CO_2_. Cells were then exposed to AlgSulf*_n_* with different DSs (*n* = DS = 0.0, 0.8, 2.0, and 2.7) and heparin as a positive control. The untreated wells in which we added only media acted as negative controls, while the vehicle wells were treated with the same volume of serum-free media as the volume used in the AlgSulf*_n_*-treated wells. Cells were treated with biomimetic sulfated GAGs at 10 or 100 µg/mL and representative bright-field images were taken 24 h after the treatment.

### 4.4. MTT Assay

Cells were plated overnight on 96-well plates—one per time point (24 h and 48 h)—at the density of 3000 and 2000 cells/well for H1792 and MDA-F471, respectively. Cells were then exposed to AlgSulf*_n_* with different DSs (DS = 0.0, 0.8, 2.0, and 2.7) and heparin as a positive control. Cells were treated with biomimetic sulfated GAGs at 10 or 100 µg/mL and analyzed at each time point after the addition of treatment. Cell metabolic activity was determined using the MTT assay (Roche). At each time point using manufacturer’s protocol, 10 µL of MTT solution was added for every 100 µL of the culture media, and the plates were incubated for 4 h in a humidified incubator. Then, we added 100 μL of stop dye solution into each well to dissolve the formazan crystals and stop the reaction. Finally, after overnight incubation, the reduced MTT optical density was measured at a wavelength of 595 nm using an ELISA reader (Multiskan FC Microplate Photometer, ThermoFisher©, Waltham, MA, USA). Cell activity was expressed as percentage growth of treated wells relative to control wells.

### 4.5. Trypan Blue Exclusion Assay

Trypan blue exclusion assay for MDA-F471 and H1792 was performed similar to MTT assay, but after 24 h and 48 h of AlgSulf*_n_* treatments, the media was replaced with 50 µL/well of trypsin/EDTA. After 5 min of incubation with trypsin/EDTA, the samples were neutralized by adding 100 µL complete media. Then, 50 µL of cell suspension was collected and mixed with 50 μL of trypan blue (Sigma-Aldrich, St. Louis, MO, USA) in an Eppendorf tube. In addition, we did trypan blue exclusion assay to assess the cell viability of the normal epithelial cells using RWPE1. Viable cells were counted on the four corner chambers of a hemocytometer and viable cell concentrations were determined by using the following formula:

cells/mL = average number of cells × dilution factor × volume of suspension (mL) × 10^4^ (1)

### 4.6. Wound Healing Assay

Wound healing assay/scratch assay was previously used to assess the directional migration of cells in vitro by mimicking the in vivo cellular migration [63]. The two cell lines were seeded on 12-well plates until they reached 90–100% confluency. Then, 2 μg/mL of mitomycin C (Sigma-Aldrich, St. Louis, MO, USA) was added to each well for 20 min to block cellular proliferation. The media was discarded, and the monolayer was scraped to make a scratch in the middle of each well using a 200 μL micropipette tip. The wells were then washed twice using phosphate-buffered saline to remove the detached cells, and the attached cells were treated with AlgSulf*_n_* 100 μg/mL concentration. Images were taken at 0, 6, 18, 24, and 48 h using bright-field Leica Application Suite software and analyzed using Zen software (version 2.5). The distance traveled by the cells represented the wound closure.

### 4.7. 3D Culture and Sphere-Formation Assay

Single H1792 and MDA-F471 cell suspensions were suspended in growth factor-reduced Matrigel™/cold serum-free DMEM (1:1) at concentrations of 2000 and 3000 cells/well, MDA-F471 and H1792 respectively, in a total volume of 50 μL in each well of a 24-well plate. Cells were seeded uniformly in a circular manner around the bottom rim of each well and allowed to solidify in the incubator at 37 °C for 45 min. Then, 500 µL of warm medium containing 5% FBS (Sigma-Aldrich, St. Louis, MO, USA) was added gently in the middle of each well and incubated in a humidified incubator (5% CO_2_) at 37 °C, following our previously established protocol on growing tumor spheres [57]. Spheres were replenished with a new fresh medium every three days. After seven days, spheres were counted and the SFU expressed as % of seeded cells, was represented by the following formula:

SFU = 100 × number of formed spheres/the number of input cells (2000 and 3000, for MDA-F471 and H1792, respectively)(2)

For sphere propagation, medium was removed and the Matrigel™ was dissolved with 0.5 mL Dispase solution (Gibco, 0.5 mg/mL, dissolved in serum-free DMEM Ham’s F-12) for 45 min at 37 °C. The released spheres were collected and centrifuged at 1200 rpm for 7 min. The pellet was suspended with 1 mL trypsin-EDTA and incubated for 6 and 12 min, respectively, for H1792 and MDA-F471, and then suspended in 1 mL media with 5% FBS to neutralize trypsin, followed by centrifugation at 900 rpm for 5 min. Cells were suspended in serum-free media and counted using a hematocytometer and then re-seeded at the same density used for each cell line in a 24-well plate in which the wells were treated with 100 µg/mL of the AlgSulf*_n_*. The media was replenished every three days. After one week of propagation, the spheres were counted, and bright-field images were taken using a Zeiss Axiovert microscope at 5× to measure the diameter and 20× to be used as representative images.

### 4.8. Total RNA Extraction

Total RNA was purified from spheres formed at G2 using RNeasy Plus kit (Qiagen, Hilden, Germany) according to the manufacturer’s protocol. Concentrations of RNA samples were quantified using the DS-11 FX spectrophotometer (DENovix, Wilmington, DE, USA) according to the manufacturer’s instructions. The purity of RNA was determined using the 260/280 ratio and a ratio of approximately 2.0 was considered as good quality RNA.

### 4.9. Two-Step Quantitative Real-Time Polymerase Chain Reaction (qRT-PCR)

Total RNA samples, from MDA-F471 and H1792 CSCs from spheres at G2 (one biological replicate), were reverse transcribed into cDNA using the QuantiTect Reverse Transcription kit (Qiagen, Hilden, Germany), according to the manufacturer’s instructions. The PCR was carried out with iTaq Universal SYBR Green Supermix (BioRad, Hercules, CA, USA) using a BioRad CFX-384 RT-PCR. The qRT-PCR thermal cycling conditions, which included a melt curve specific for the product, are summarized in Table S1. We analyzed the obtained data using the 2^−∆∆Ct^ calculation method by normalizing to two conserved reference genes: Glyceraldehyde 3-phosphate dehydrogenase (*GAPDH*) and TATA-box binding protein (*TBP*), in which these two genes were suggested in the expression analysis of CSCs’ genes [58]. Gene expression of *ALDH1A1*, *CCL20*, and *ALDH3A1* in H1792 and *Ccl20* and *Tnf* in MDA-F471 were normalized to human *GAPDH* and murine *Gapdh,* respectively, while that of Aldh1a1 and *Alcam* in MDA-F471 were normalized to *Tbp*. The gene expression of these CSC-markers was computed and analyzed with respect to the control. Each reaction was done only once with technical duplicates. The assessed human and murine primer sequences, as well as their annealing temperatures, are indicated in Tables S2 and S3, respectively.

### 4.10. Statistical Analysis

Statistical analysis was done using GraphPad Prism 7 analysis software (version 7.00, GraphPad, San Diego, CA, USA). The significance of the data was analyzed using one-way ANOVA or two-way ANOVA, followed by Bonferroni’s multiple comparison test. Differences were considered statistically significant for *p*-values less than 0.05. (* *p* < 0.05; ** *p* < 0.01; *** *p* < 0.001). A *p*-value of *p* < 0.001 was considered highly significant.

## 5. Conclusions

In conclusion, this is the first study that systematically assesses the effect of the sulfation of biomimetic sulfated GAGs on *Kras* mutant LUAD cell lines. The increase in the sulfation of the biomimetic GAGs had inhibitory effects on the proliferation of murine *Kras*-mutant LUAD, though not as pronounced in the human cell lineage. An effect on the cell viability was also evident in the murine cell line, as well as the human cell line at higher DS. The biomimetic sulfated GAGs also had a significant effect on cell migratory abilities for both cell lineages. In addition to the effects assessed in 2D assays, there was also significant effects on the sphere forming abilities as well as sphere sizes formed in 3D assays. These results pave the way for future studies to assess the effects of the increase in the DS of mimetic GAGs on the expression of CSCs markers and in vivo LUAD models towards the assessment of these materials for LUAD treatment.

## Supplementary Materials

The following are available online. Figure S1: The method used to prepare the sulfated alginate biomimetic GAGs. Alginate was mixed with DOWEX charged with tetrabutyl ammonium bromide, and then mixed with different ratios of SO_3_/Pyridine to achieve different DS, followed by dimethyl formamide (DMF) and ethanolic NaOH. Figure S2: The effect of the DS of biomimetic sulfated GAGs on the morphology of human and murine LUAD cells. Representative bright-field images of (**A**) H1792 cells treated with 10 μg/mL of AlgSulf*_n_*, (**B**) H1792 cells treated with 100 μg/mL of AlgSulf*_n_*, (**C**) MDA-F471 cells treated with 10 μg/mL of AlgSulf*_n_* and (**D**) MDA-F471 cells treated with 100 μg/mL of AlgSulf*_n_*. Scale bar = 100 μm. Figure S3: The effect of the DS of biomimetic sulfated GAGs on the number of live RWPE1 cells (**A**) RWPE1 treated with 10 μg/mL of AlgSulf*_n_*, (**B**) RWPE1 treated with 100 μg/mL of AlgSulf*_n_*. Results showed that the increase in the sulfation of GAGs did not affect the number of live RWPE1 normal cells. Data represent the average of three independent experiments and are reported as mean ± SEM (**p* < 0.05) using two-way ANOVA followed by Bonferroni’s multiple comparison test. Figure S4: Representative bright-field images of (**A**) H1792 and (**B**) MDA-F471 wounds with different AlgSulf*_n_*. Scale = 100 μm. Figure S5: Differential expression of selected stemness markers in human and murine spheres by qRT-PCR. (**A**) Downregulation of *ALDH1A1*, *ALDH3A1*, and *CCL20* in H1792 spheres at G2, (**B**) downregulation of *Alcam*, *Aldh1a1*, *Ccl20* and *Tnf* in MDA-F471 spheres at G2. Results were validated by qRT-PCR and analyzed using the 2^−ΔΔCt^ calculation by normalization to two different conserved reference genes (*GAPDH* for H1792) and (*Gapdh* and *Tbp* for MDA-F471) and are represented as mean ± SEM (*n* = 1). Table S1: Thermal cycling conditions of qRT-PCR. Table S2: Primer sequences and annealing temperature of some selected human genes. Table S3: Primer sequences and annealing temperature of some selected murine genes.
