# Supplementary material for: Anti-Tumor Effects of Biomimetic Sulfated Glycosaminoglycans on Lung Adenocarcinoma Cells in 2D and 3D In Vitro Models
Source: Molecules. 2020 Jun 3;25(11):2595. doi: 10.3390/molecules25112595 (PMC7321182; doi:10.3390/molecules25112595)
Supplement: Supplementary file 1 [file molecules-25-02595-s001.pdf]

Supporting Information

1. **Sulfated alginate preparation and characterization**

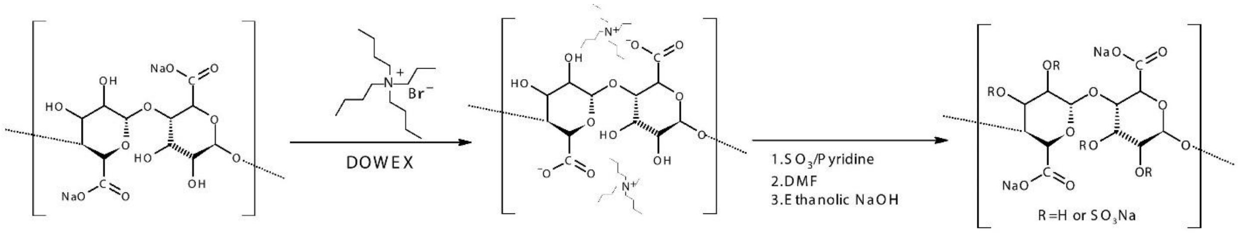

Figure S1. The method used to prepare the sulfated alginate biomimetic GAGs. Alginate was mixed with DOWEX charged with tetrabutyl ammonium bromide, and then mixed with different ratios of SO_3/Pyridine to achieve different DS, followed by dimethyl formamide (DMF) and ethanolic NaOH.

2. **Effect of the increase in the DS of sulfated GAGs on the morphology of H1792 and MDA-F471 in 2D cultures**

The images were taken using a Zeiss Axiovert microscope at 10x and analyzed using Carl Zeiss Zen 2012 image software. The images show that there is no effect on the morphology of H1792 and MDA-F471 with the increase in the sulfation of $AlgSulf_n$ (Supporting Figure S2A-D).

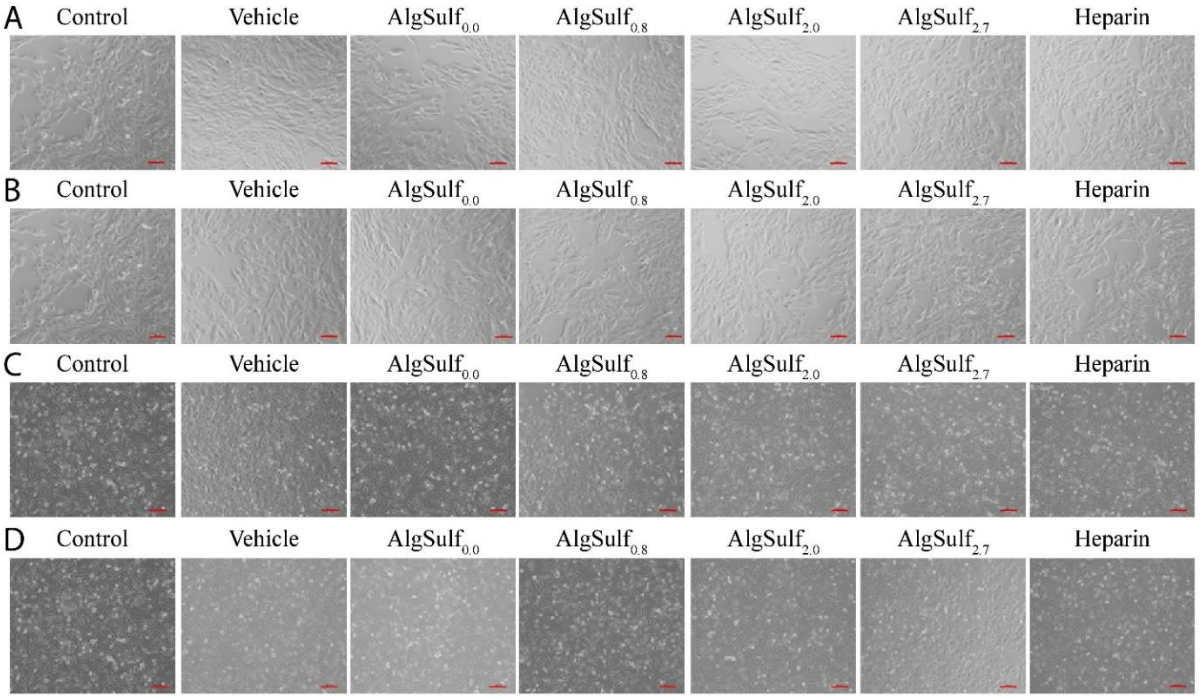

Figure S2. The effect of the DS of biomimetic sulfated GAGs on the morphology of human and murine LUAD cells. Representative bright-field images of (A) H1792 cells treated with 10 μg/mL of $AlgSulf_n$ (B) H1792 cells treated with 100 μg/mL of $AlgSulf_n$ (C) MDA-F471 cells treated with 10 μg/mL of $AlgSulf_n$ and (D) MDA-F471 cells treated with 100 μg/mL of $AlgSulf_n$. Scale bar= 100 μm.

3. **The increase in the DS of biomimetic GAGs modulates the viability of RWPE1 normal epithelial prostatic cell line in 2D culture**

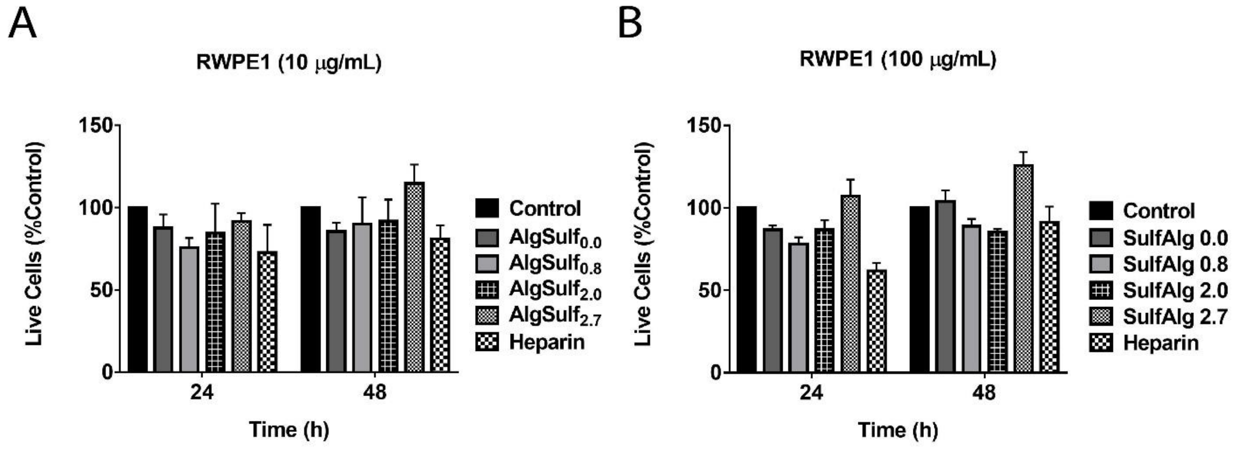

Figure S3. The effect of the DS of biomimetic sulfated GAGs on the number of live RWPE1 cells (A) RWPE1 treated with 10 µg/mL of AlgSulf$_n$ (B) RWPE1 treated with 100 µg/mL of AlgSulf$_n$. Results showed that the increase in the sulfation of GAGs did not affect the number of live RWPE1 normal cells. Data represent the average of three independent experiments and are reported as mean ± SEM (*$p < 0.05$) using two-way ANOVA followed by Bonferroni's multiple comparison test.

4. **The increase in the sulfation of biomimetic GAGs inhibits the migratory abilities of LUAD**

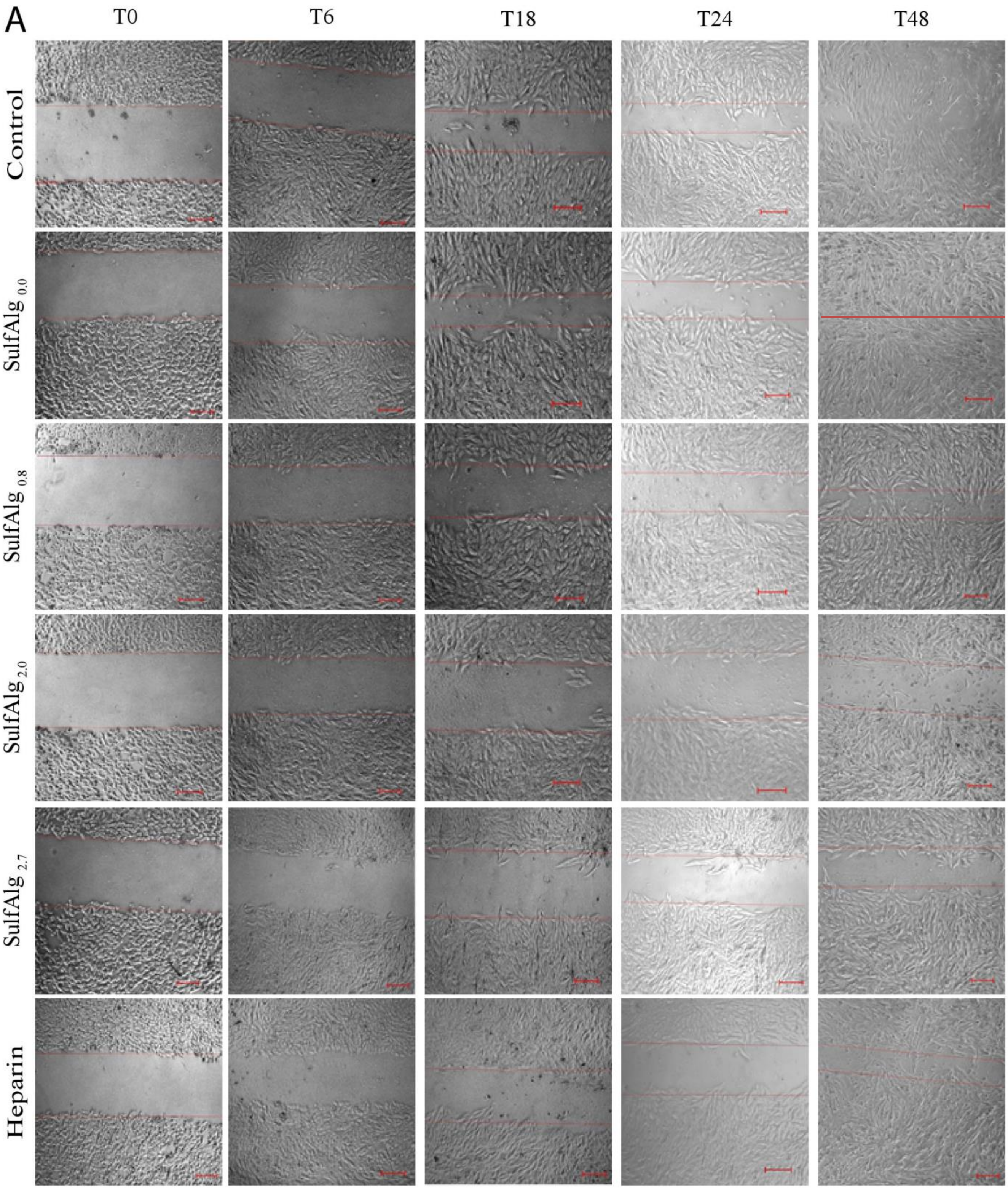

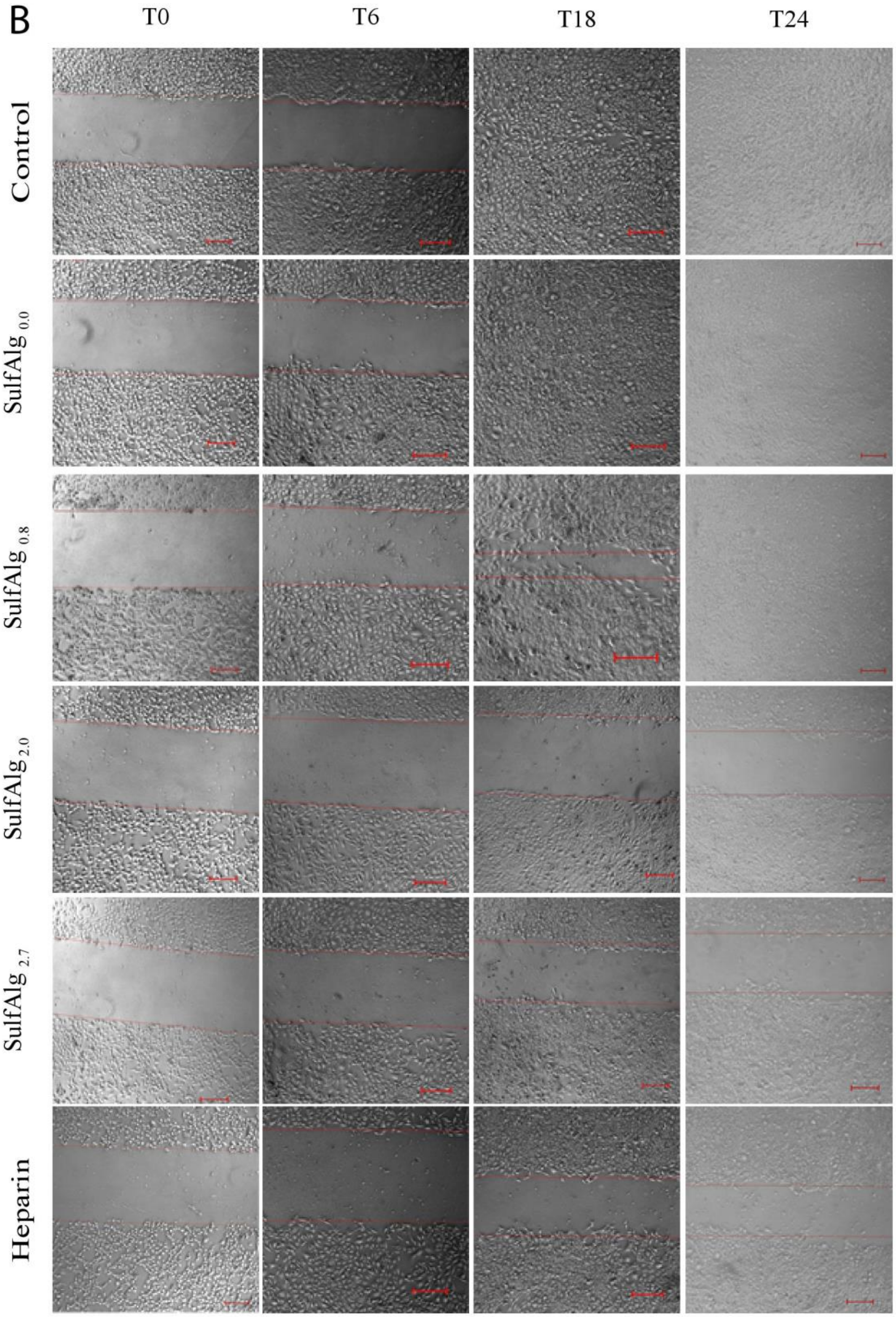

Figure S4. Representative bright-field images of (A) H1792 and (B) MDA-F471 wounds with different AlgSulf$_n$. Scale= 100 μm.

5. **The increase in the DS of AlgSulf$_n$ down-regulates the expression of some stem-cell marker genes in 3D spheres derived from LUAD cell lines using two-step quantitative real-time Polymerase Chain Reaction (qRT-PCR)**

Table S1. Thermal cycling conditions of qRT-PCR.

**Step**	**Polymerase Activation**	**Amplification (40cycle)**			**Melt Curve**
		**Denaturation**	**Annealing**	**Extension**	
Temperature (^oC)	95	95	Variable	72	65-95 in 0.5 increments
Time	5min	15sec	30sec	30sec	5sec/step

Table S2. Primer sequences and annealing temperature of some selected human genes.

**Human gene**	**Primer Sequence (5′-3′)**	**Annealing Temp.(^oC)**	**Reference**
ALDH1A1	F-TGTTAGCTGATGCCGACTTG	60	[49]
	R-ATTCTTAGCCCGCTCAACACT		
ALDH3A1	F- GCAGACCTGCACAAGAATGA	60	[50]
	R-TGTAGAGCTCGTCCTGCTGA		
CCL20	F-GGTGAAATATATTGTGCGTCTCC	60	[51]
	R-ACTAAACCCTCCATGATGTGC		
GAPDH	F-GGACCTGACCTGCCGTCTA	60	[52]
	R- TGGTGCTCAGTGTAGCCCAG		

Table S3. Primer sequences and annealing temperature of some selected murine genes.

**Murine gene**	**Primer Sequence (5′-3′)**	**Annealing Temp.(^oC)**	**Reference**
Alcam	F-ATGGCATCTAAGGTGTCCCCT	60	[49]
	R-AGACGGCAAGGCATGACAA		
Aldh1a1	F-GACAGGCTTTCCAGATTGGCTC	60	[50]
	R-AAGACTTTCCCACCATTGAGTGC		
Ccl20	F-GTGGGTTTCACAAGACAGATG	57	[53]
	R-TTTTCACCCAGTTCTGCTTTG		
Gapdh	F-GCAAAGTGGAGATTGTTGCCA	60	[54]
	R-GCCTTGACTGTGCCGTTGA		
Tbp	F-CCTTGTACCCTTCACCAATGAC	60	[55]
	R-ACAGCCAAGATTCACGGTAGA		
Tnf	F-TCAGCCGATTTGCTATCTCATA	57	[56]
	R-AGTACTTGGGCAGATTGACCTC		

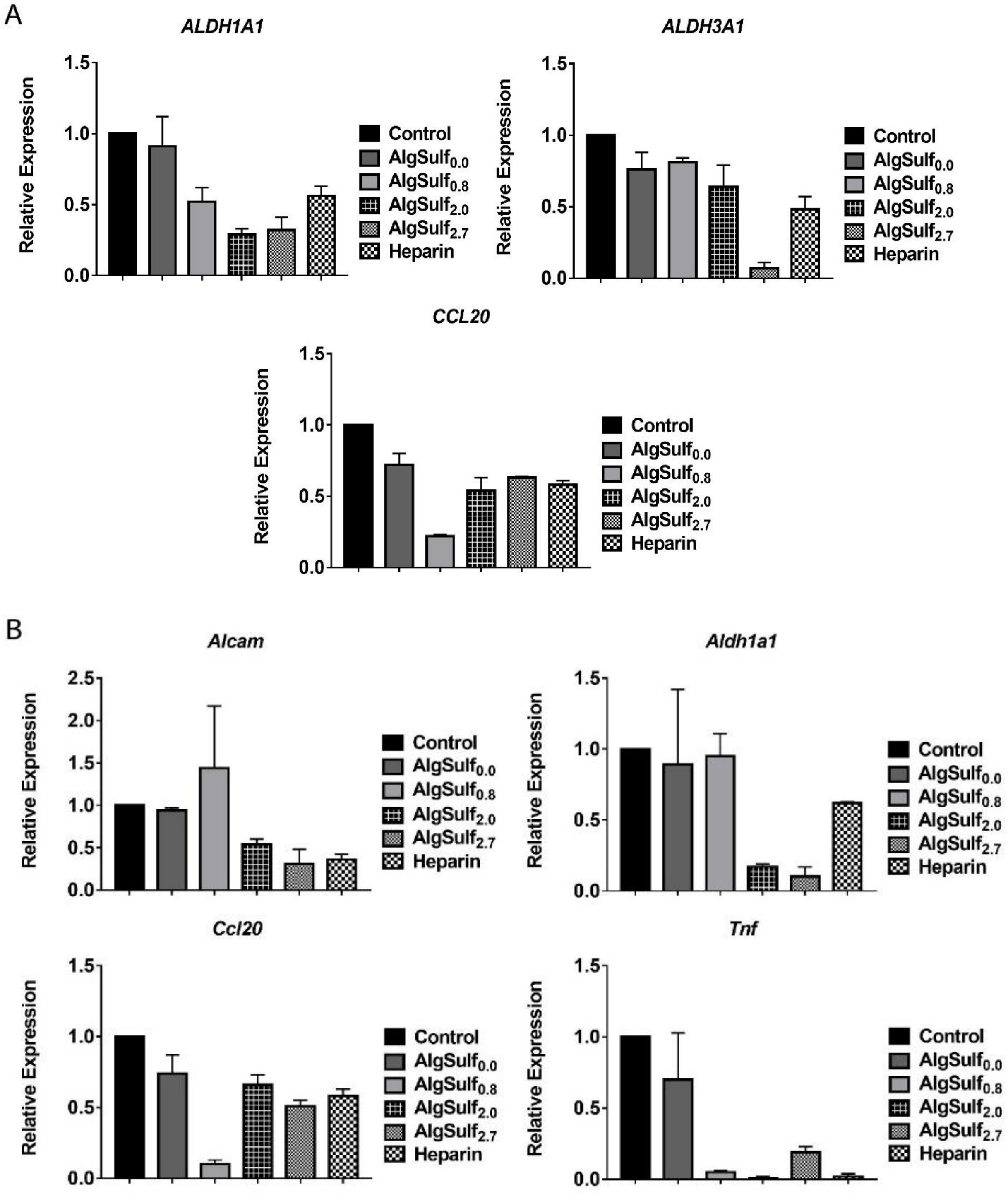

Figure S5. Differential expression of selected stemness markers in human and murine spheres by qRT-PCR. (**A**) Downregulation of *ALDH1A1*, *ALDH3A1*, and *CCL20* in H1792 spheres at G2 (**B**) downregulation of *Alcam*, *Aldh1a1*, *Ccl20* and *Tnf* in MDA-F471 spheres at G2. Results were validated by qRT-PCR and analyzed using the $2^{-\Delta\Delta Ct}$ calculation by normalization to two different conserved reference genes (*GAPDH* for H1792) and (*Gapdh* and *Tbp* for MDA-F471) and are represented as mean ± SEM (n=1).
